# Supplementary material for: How and when doula support increases confidence in women experiencing socioeconomic adversity: Findings from a realist evaluation of an Australian volunteer doula program
Source: PLoS One. 2022 Jun 30;17(6):e0270755. doi: 10.1371/journal.pone.0270755 (PMC9246160; doi:10.1371/journal.pone.0270755)
Supplement: Attachment — Submitted filename: Response to Reviewers.docx [file pone.0270755.s001.docx]

28 April 2022

Professor Sharon Mary Brownie
Academic Editor

PLOS ONE

**Re: Submitted manuscript *“How and when doula support increases confidence in women experiencing socioeconomic adversity: findings from a realist evaluation of an Australian volunteer doula program”.***

Dear Professor Brownie,

Thank you for the reviews of this manuscript. The manuscript has been modified and below are our responses to the specific points made by you and the reviewers.

Thank you for considering this paper for publication, and please let me know if any further clarification is required.

Yours sincerely,

Kerryn O’Rourke, and

Jane Yelland

Michelle Newton

Touran Shafiei

|  | **REQUIREMENTS/COMMENTS** | **RESPONSE** | **MODIFICATION TO MANUSCRIPT**  **(HIGHLIGHTED IN YELLOW)** |
| --- | --- | --- | --- |
| **Journal requirements** |  |  |  |
| **a.** | Please ensure that your manuscript meets PLOS ONE's style requirements, including those for file naming. | Some slight modifications to heading styles have been made in accordance with this style guide.  All level 2 headings were increased to 14-point font. | See manuscript. |
| **b.** | In your Data Availability statement, you have not specified where the minimal data set underlying the results described in your manuscript can be found.  If there are ethical or legal restrictions on sharing a de-identified data set, please explain them in detail (e.g., data contain potentially sensitive information, data are owned by a third-party organization, etc.) and who has imposed them (e.g., an ethics committee). Please also provide contact information for a data access committee, ethics committee, or other institutional body to which data requests may be sent. | The sources of data reported in this study cannot be shared openly out of respect for the privacy of participants stipulated in the Human Research and Ethics Committee’s (HREC) approval. Data requests should be made to La Trobe University HREC (study HEC19388) at humanethics@latrobe.edu.au | N/A |
| **c.** | Please review your reference list to ensure that it is complete and correct. If you have cited papers that have been retracted, please include the rationale for doing so in the manuscript text, or remove these references and replace them with relevant current references. Any changes to the reference list should be mentioned in the rebuttal letter that accompanies your revised manuscript. If you need to cite a retracted article, indicate the article’s retracted status in the References list and also include a citation and full reference for the retraction notice. | One reference removed for blinding has now been fully cited.  This is also addressed in response to reviewer 2, below. | 23. O’Rourke KM, Yelland J, Newton M, Shafiei T. An Australian doula program for socially disadvantaged women: Developing realist evaluation theories. Women and Birth. 2019;33(5):e438-e46. doi: <https://doi.org/10.1016/j.wombi.2019.10.007>. |
| **Reviewer 1** |  |  |  |
| **1.1** | This is a useful article, which can bring a new insight into the care of a new mother of disadvantaged group. The authors have skill fully analysed COM in community-based volunteer doula program by realist evaluation. The methodology, data analysis and presentation is done clearly. My recommendation is to publish this article. | Thank you for this feedback. | N/A |
| **Reviewer 2.** |  |  |  |
| **2.1** | Thank you for allowing me to review this very interesting paper about how and why doula support to the birth process may strengthen confidence. It is refreshing to read about strategies to effectively change and improve delivery of care to pregnant people. This is a well written manuscript. I have only a few comments. | Thank you for this feedback. Please see the below responses and respective modifications made to the manuscript in the next column. | N/A |
| **2.2** | The [PLOS Data policy](https://protect-au.mimecast.com/s/JBikCk8vz8cZyDxxI0X-ty?domain=plosone.org) requires authors to make all data underlying the findings described in their manuscript fully available without restriction, with rare exception (please refer to the Data Availability Statement in the manuscript PDF file). The data should be provided as part of the manuscript or its supporting information, or deposited to a public repository. For example, in addition to summary statistics, the data points behind means, medians and variance measures should be available. If there are restrictions on publicly sharing data—e.g. participant privacy or use of data from a third party—those must be specified. | Please refer to journal requirement (b) above. | N/A |
| **2.3** | Pre-post data is referred to in the manuscript, eg line 36 in abstract - I'm curious to know if this pre and post program, or pre and post delivery. At exactly what time points were the pre and post data collected? | These are pre and post program support data. The time points are described in the Methods – Data collection section, at lines 201-204. The word ‘support‘ has been added for clarity. | *The pre and post support birth confidence measures used in this study were women’s answers to two questions asked by telephone, using interpreters where required. The first was at the time of program intake by a program coordinator (How confident do you feel about giving birth?), and then followed up at three to six months after birth (How confident did you feel about your birth?), by a program coordinator, contracted bicultural worker or administration assistant.* |
| **2.4** | Introduction, line 49 - I think relational care with continuity could be provided by any practitioner - would just remove (usually a midwife). | As suggested, ‘(usually a midwife)’ has been removed. | *It is well established that the best maternity care outcomes are achieved by relational care with continuity—provided by the same caregiver over time [1, 2].* |
| **2.5** | Line 88, reference is [x] - is a numbered reference missing? | The X indicated a reference removed for blinding as it is by the same authors. The reference has now been added. | *The theories and their development are published elsewhere [23].*  23. O’Rourke KM, Yelland J, Newton M, Shafiei T. An Australian doula program for socially disadvantaged women: Developing realist evaluation theories. Women and Birth. 2019;33(5):e438-e46. doi: <https://doi.org/10.1016/j.wombi.2019.10.007>. |
| **2.6** | Line 90 - since this manuscript is only dealing with. the "being by her side" theory, I'd recommend removing the reference to other theories not evaluated here. | The manuscript situates this study within a broader study of four theories by stating “*Four theories were prioritised for the evaluation”.* We would prefer to leave this reference as it indicates that other theories were also prioritised in the evaluation of a complex program. | The following text is unchanged:  *Four theories were prioritised for the evaluation. This paper reports the testing and refinement of one of these theories, called Being by her side.*  *(Lines 91-92).* |
| **2.7** | Data collection. Did you have a pre-defined set of context, outcomes, or possible mechanisms? Or were these solely defined by the interviews?  Since 'elements of CMO's were put to women' (line 112), I'm wondering if a chart of predetermined contexts, mechanisms, and/or outcomes could be included here. This would help the reader understand exactly what was assessed and then not included in the final models. | Thank you. We are a little unclear about this comment.  The hypothesised program theory was developed from key informant interviews and a rapid realist review of literature, as described in the Introduction, at lines 84-89. The theory itself, comprised of CMO configurations, is then described at lines 91-101. We would be happy to convert this to table format for clarification if required. | The following text is unchanged:  *This paper reports the testing and refinement of one of these theories, called Being by her side.* *Comprised of two CMO configurations, the theory hypothesised how, when and for whom doula support causes a woman (client) to feel dignified and confident. The first CMO suggested that when a woman is experiencing socioeconomic disadvantage (context) and is well matched with her doula (context), the doula is ‘by her side’, ‘gets’ her, gives time, attention and respect, and believes in her (resource). In response, the woman sees her own strength and value (reasoning), and this leads to feeling dignified and confident (outcome). The second CMO hypothesised that when a doula is kind and non-judgemental (context), she notices a woman’s small wins, and praises the woman as a mother (resource). In response, the woman feels validated as a mother (reasoning) and then dignified and confident (outcome).*  *(Lines 91-101).* |
| **2.8** | Did you have any information about other birth support people? (family, friend, partner...). Could other support people in her life be a mediator in these models, or lack thereof possibly explain some of the decline in confidence. I'm wondering if this is important during doula visits as well as at the time of birth. Could other support people pick up on cues from the doula and hence support or interfere. | Thank you.  No association was found between a women’s relationship status (partnered or unpartnered) and birth confidence in the pre-post support quantitative data. It is possible that the presence or absence of other social support in a woman’s life (eg. family, friends) could act as a context affecting the way in which doula support works. A line about this has been added to the Discussion section. | *Future iterations of evaluation could build on the findings of this study by asking interview participants for permission to access their routinely collected data. Linking qualitative data on contexts and mechanisms with quantitative outcome data may enable stronger explanations of outcomes (birth confidence changes). Targeted recruitment for qualitative theory testing with women who do not have increased confidence and either continue in the program or withdraw, and doulas who have left the program, may also build on findings. Exploration of new hypotheses including different attachment styles of women as contexts enabling/preventing trust with doulas, mother-baby attachment, and other long-term outcomes, would further refine the program theory. Exploration of the effects of other social supports (eg. friends or family) as contexts affecting how doula support works may be valuable as well.*  *(Lines 573-574).* |
